# Supplementary figures and images for: Plasma BRAF Mutation Detection for the Diagnostic and Monitoring Trajectory of Patients with LDH-High Stage IV Melanoma
Source: Cancers (Basel). 2021 Aug 3;13(15):3913. doi: 10.3390/cancers13153913 (PMC8345527; doi:10.3390/cancers13153913)

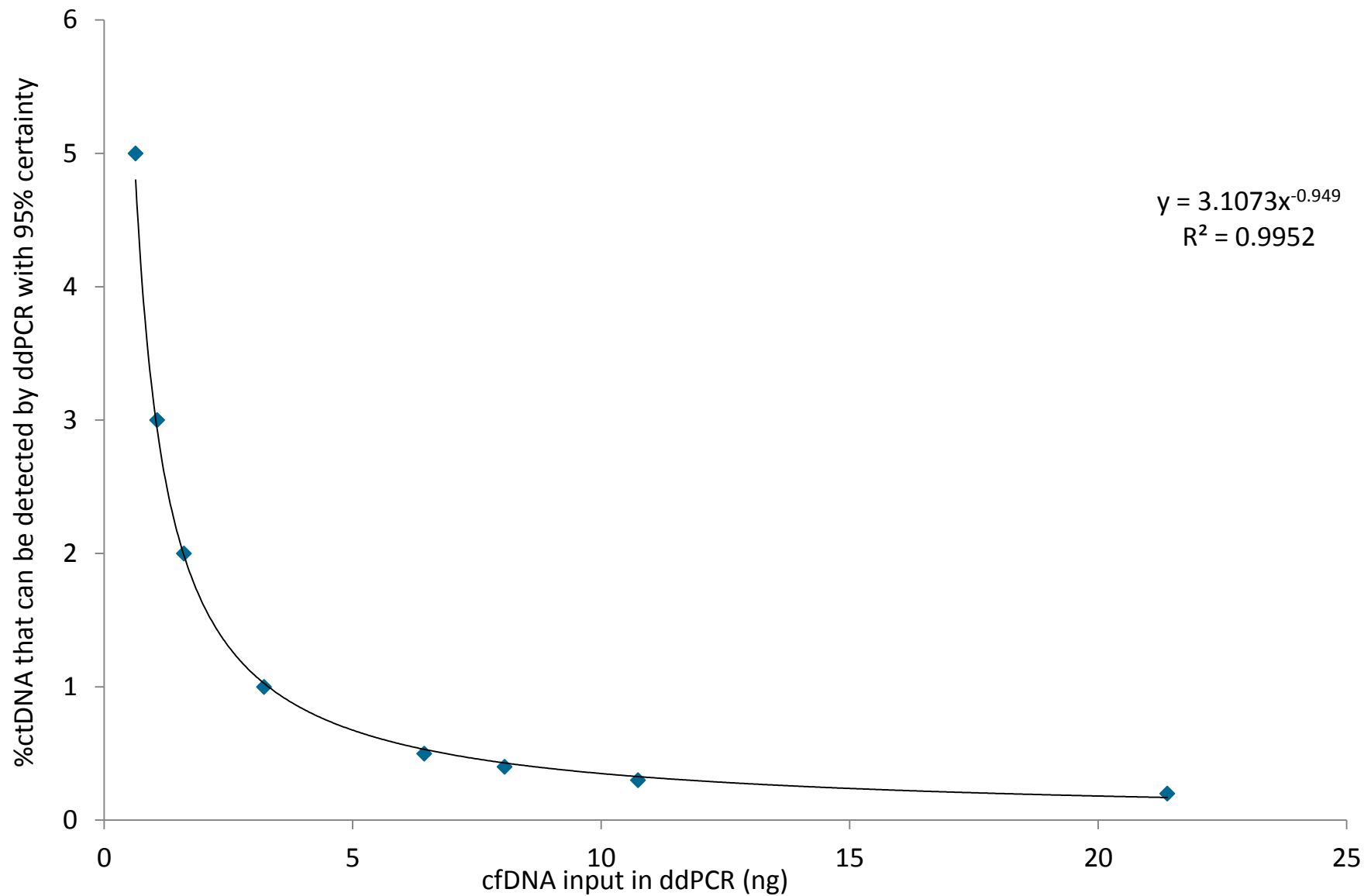

Supplement: Supplementary file 1 [file cancers-13-03913-s001.zip › Supplementary Data/20210625 Supplementary Figure S1.pdf]

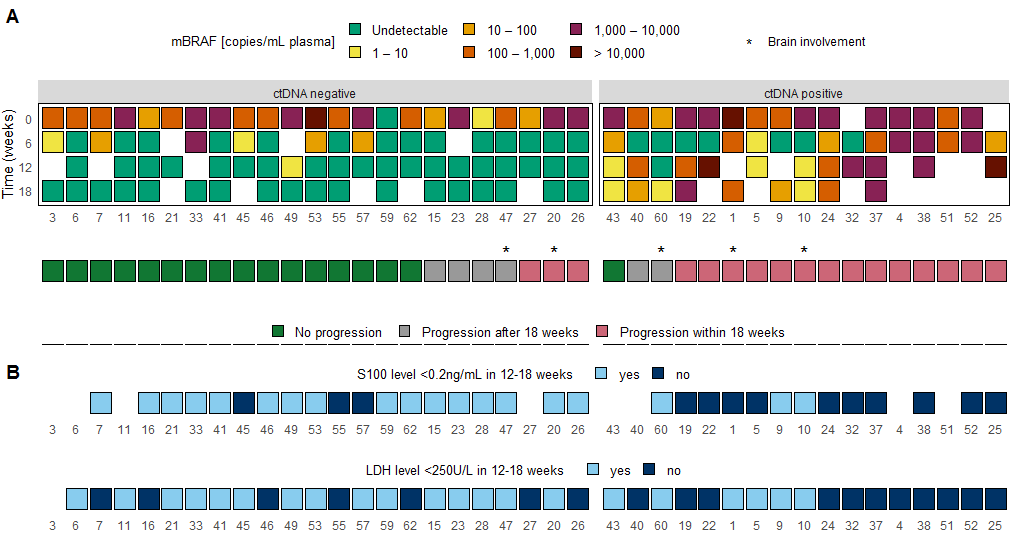

Supplement: Supplementary file 1 [file cancers-13-03913-s001.zip › Supplementary Data/20210802 Suppl Figure 2.tiff]
